# Intravitreal Administration Effect of Adipose-Derived Mesenchymal Stromal Cells Combined with Anti-VEGF Nanocarriers, in a Pharmaceutically Induced Animal Model of Retinal Vein Occlusion

**DOI:** 10.1155/2022/2760147

**Published:** 2022-02-23

**Authors:** Eleni Gounari, Anastasia Komnenou, Evangelia Kofidou, Stavroula Nanaki, Dimitrios Bikiaris, Stavroula Almpanidou, Kokkona Kouzi, Vasileios Karampatakis, George Koliakos

**Affiliations:** ^1^Department of Biochemistry, School of Medicine, Aristotle University of Thessaloniki, Thessaloniki, Greece; ^2^Biohellenika Biotechnology Company, Thessaloniki, Greece; ^3^Laboratory of Experimental Ophthalmology, School of Medicine, Aristotle University of Thessaloniki, Thessaloniki, Greece; ^4^School of Veterinary Medicine, Aristotle University of Thessaloniki, Thessaloniki, Greece; ^5^Department of Biological Applications and Technology, University of Ioannina, Greece; ^6^Department of Chemistry, Laboratory of Polymer Chemistry and Technology, Aristotle University of Thessaloniki, Thessaloniki, Greece; ^7^Department of Histology Embryology, School of Medicine, Aristotle University of Thessaloniki, Thessaloniki, Greece

## Abstract

Antiangiogenic therapeutic agents (anti-VEGF) have contributed to the treatment of retinal vein occlusion (RVO) while mesenchymal stromal cell- (MSCs-) mediated therapies limit eye degeneration. The aim of the present study is to determine the effect of adipose-derived MSCs (ASCs) combination with nanocarriers of anti-VEGF in a pharmaceutically induced animal model of RVO. Nanoparticles (NPs) of thiolated chitosan (ThioCHI) with encapsulated anti-VEGF antibody were prepared. ASCs were isolated and genetically modified to secrete the green fluorescence GFP. Twenty-four New Zealand rabbits were divided into the I-IV equal following groups: ASCs, ASCs + nanoThioCHI-anti-VEGF, RVO, and control. For the RVO induction, groups I-III received intravitreal (iv) injections of MEK kinase inhibitor, PD0325901. Twelve days later, therapeutic regiments were administered at groups I-II while groups III-IV received BSS. Two weeks later, the retinal damage evaluated via detailed ophthalmic examinations, histological analysis of fixed retinal sections, ELISA for secreted cytokines in peripheral blood or vitreous fluid, and Q-PCR for the expression of related to the occlusion and inflammatory genes. Mild retinal edema and hemorrhages, limited retinal detachment, and vasculature attenuation were observed in groups I and II compared with the pathological symptoms of group III which presented a totally disorganized retinal structure, following of positive immunostaining for neovascularization and related to RVO markers. Important reduction of the high secreted levels of inflammatory cytokines was quantified in groups I and II vitreous fluid, while the expression of the RVO-related and inflammatory genes has been significantly decreased especially in group II. GFP+ ASCs, capable of being differentiated towards neural progenitors, detected in dissociated retina tissues of group II presenting their attachment to damaged area. Conclusively, a stem cell-based therapy for RVO is proposed, accompanied by sustained release of anti-VEGF, in order to combine the paracrine action of ASCs and the progressive reduction of neovascularization.

## 1. Introduction

Retinal vein occlusion (RVO) is the second leading cause of retinal vascular disease after diabetic retinopathy (DR) [1]. There are two broadly accepted types of RVO: central retinal vein occlusion (CRVO), when the central vein of the retina is affected, and branch retinal vein occlusion (BRVO), when any of the branches of the central retina vein is occluded. BRVO has been reported to be three times more common than CRVO [2]. Underlying mechanisms of the vision loss in patients with RVO include retinal ischemia and macular edema [3]. Retinal vascular diseases constitute a major healthcare problem in ophthalmology due to the devastating effects on the visual performance of affected individuals, precluding them from performing their daily activities and leading to a severe reduction of their quality of life (QoL) [4–6].

Early diagnosis of retinal abnormalities related to RVO is fundamental for the clinical outcome of the affected individuals. Microvascular changes in both the superficial and deep capillary networks of the retina such as a decrease in foveal and parafoveal vascular densities, capillary engorgement and telangiectasias, nonperfusion area microaneurysms, and formation of collateral vessel can be detected in retinas with vein occlusions using the optical coherence angiography (OCTA) [7]. Swept-source optical coherence tomography angiography (SS-OCTA) has found to be more precise in defining the areas of maximum ischemic injury following RVO compared to fundus fluorescein angiography (FFA), hence represents a significant diagnostic device in predicting the visual outcome [8].

Regarding the current therapeutic approach of the RVO, intravitreal injections of antivascular endothelial growth factor (anti-VEGF) agents are commonly used to improve the clinical outcomes in RVO patients [9]. There is also growing evidence supporting the use of intravitreal dexamethasone implants in patients with retinal vascular diseases, especially in those with macular edema, at the expense an elevation in intraocular pressure and cataract progression or even formation, side effects typically associated with steroids [3, 10–14]. Furthermore, severe side effects of anti-VEGF intravitreal injections including endophthalmitis and retinal detachment and the observation that some eyes become refractory after repeated injections are strong disadvantages making this type of therapy partially effective [15].

A delivery system capable of continuous and long-term release of anti-VEGF could potentially overcome the above limitations.

Nanoparticles have been widely used as drug delivery systems due to their ability to efficiently deliver the incorporated substances with reduced side effects to the patient. One of the most studied polymer in multiple applications is chitosan, due to its properties such as biocompatibility, biodegradability, and low toxicity [16–18]. The increase of its mucoadhesiveness can be achieved with the modification of its structure after the addition of thiol groups which acts as cell receptors able to facilitate cell adhesion, proliferation, and differentiation [19–21]. The delivery of antibodies from chitosan nanoparticles constitutes a new area on their usage making the role of this polymer even more important. Taking into consideration all the previously mentioned, we have recently developed a novel nanocarrier, constructed of thiolated chitosan, able to gradually release anti-VEGF for at least one week in cell cultures [22].

Mesenchymal stromal cells (MSCs) and related to them components consist another promising approach for several eye disorders and especially for RVO treatment especially due to their paracrine action which enables ganglion cells' protection limiting further degeneration of the eye [23–27]. MSCs present neuroprotective effects on degenerated retinal cells, which could be associated with delaying or even stopping of uncontrolled cell death [28, 29].

Adipose-derived mesenchymal stromal cells (ASCs) can be easily isolated following a noninvasive procedure during liposuction. As the majority of stem cells, ASCs have high proliferative capacity while they are able to be differentiated towards cells from other germ layers, such as neural and retinal cells [30, 31]. In addition, ASCs can perform their paracrine action by secreting many cytokines and growth factors directly or throughout exosome release in order to enforce the regeneration of damaged tissues [32]. All the above, make ASCs a proper cellular product for treatment of several retinopathies. The findings from several clinical trials have shown that some patients who received intravitreal transplantation of human ASCs experienced dense vitreous hemorrhage and retinal detachments following the subsequent development of proliferative vitreoretinopathy (PVR). These complications have also been associated with the surgical procedure, the underlying mechanism of ASCs' action upon injection, the time of transplantation, or the number of administered cells and are still a significant issue of constant research [33, 34]. The ease of obtaining and preparing these cells makes their application really attractive to clinicians and researchers.

The inhibitor MEK 1/2 PD0325901 or N-[(2R)-2,3-dihydroxypropoxy]-3,4-difluoro-2-[(2-fluoro-4-iodophenyl)amino]-benzamide] has been widely applied for solid tumor therapies. However, one of the main negative outcomes reported by all the registered clinical trials using PD0325901 is the production of RVO with the appearance of all the main clinical symptoms of the disease. Exploiting this side effect, researchers have already developed an in vivo model of RVO by applying intravitreal injections of the inhibitor to Dutch-Belted rabbits [35]. Although several animal models exist, the development of an easily induced animal model of RVO is still needed to improve current understanding of the pathogenesis of RVO, as well as to identify more clinically effective and cost-effective therapeutic options [36].

Based on all the above, the aim of the present study is to determine the effect of intravitreal administration of ASCs combined with encapsulated anti-VEGF in thiolated chitosan nanocarriers (nanoThioCHI) on a polyparametrically characterized PD0325901-mediated induced animal model of RVO.

## 2. Methods

### 2.1. Study Approval

All procedures were approved by the local Ethics Committee of the Aristotle University of Thessaloniki (4.296-4/26.1.2021), as well as by the Committee of the Department of Veterinary Medicine of Thessaloniki (661411(2802)/3.12.2020) and conformed to the ARVO Statement for the Use of Animals in Ophthalmic and Vision Research and the European Communities Council Directive (86/609/EEC).

### 2.2. Isolation, Expansion, and Characterization of Rabbit ASCs

6-month-old New Zealand rabbits weighing 3.5-4 kg were anaesthetized with 0.08-0.1 mg/kg b.w. dexmedetomidine (Dexdomitor, Zoetis Hellas) and 15 mg/kg b.w. ketamine (Imalgene 1000, Merial, France) intramuscularly. *Τ*he inguinal fat pad was obtained, washed with phosphate-buffered saline (PBS; BIOWEST), and minced and digested with 0.5 mg/ml collagenase type-1 (Sigma) for 1 h at 37°C with constant shaking. PBS was added, and 30 min later, the distinct middle layer containing MSCs was aspirated and centrifuged for 10 min at 600 × g. The cellular pellet was resuspended in Dulbecco's modified Eagle's medium (DMEM; BIOWEST) supplemented with 5% fetal calf serum (FBS; BIOWEST) and 1% penicillin-streptomycin (pen-strep; Sigma), and cells were cultured to 80% confluency in 75 cm^2^ tissue culture flasks in an incubator at 37°C with 5% CO2 [37]. Between passages 2 and 3, the cells were characterized via flow cytometry upon staining with monoclonal antibodies CD105/CD73-phycoerythrin (PE) and CD90/CD44-fluorescein (FITC) (EXBIO). The BD FACSCalibur (Becton Dickinson, BD) flow cytometer and the CellQuest Pro6 software were used for result analysis.

To test the differentiation capacity of ASCs, the appropriate medium (Gibco) for induced differentiation towards osteocytes, adipocytes, and chondrocytes was added in the culture for 25-30 days accompanied with media changes every 2-3 days. The success of differentiation was estimated with alizarin red, oil red, and alcian blue staining, respectively, according to each differentiation medium manufacturer's instructions.

### 2.3. Genetic Modification of ASCs

On passage 4, 1.5∗10^5^ cells were exposed to 10 *μ*g of plasmid DNA-SB100X transposase and pT2-Venus-neo transposon expression plasmids (1: 10 ratio) and were electroporated according to the manufacturer's instructions (Lonza). The cells were then plated in one well of a 6-well plate in the presence of DMEM full medium until reaching a 90% confluency, whereas 100 mg/ml G418 (InvivoGen) was added for the selection of the genetically modified ASCs. The cells were incubated in 37°C with 5% CO2 and were subjected to media changes every 2–3 days. The successful modification was assessed with fluorescence microscopy HBO 50 mercury lamp as well as reflectors with fluorescence filter (excitation 488 nm, emission 509 nm) and Fluorescence Lite software module of AxioVision LE (Carl Zeiss). Flow cytometry was performed to determine the percentage of genetic modified ASCs.

### 2.4. Preparation of Thiolated Chitosan (ThioCHI) NPs Containing Anti-VEGF and Characterization

Nanoparticles of ThioCHI containing anti-VEGF were prepared by ionic gelation technique as was previously described [21, 22]. 200 mg of ThioCHI was dissolved in 25 ml of aqueous solution of acetic acid and 1% *v*/*v* in concentration. TPP aqueous solution, 2 mg/ml in concentration, and 25 ml in volume were added dropwise in ThioCHI solution under continuous magnetic stirring, having final ratio ThioCHI/TPP 4/1. 50 *μ*g of anti-VEGF was dispersed in water and added to the solution right before the addition of TPP solution, while the pH was immediately adjusted to pH = 7 right after its addition to the solution. The total solution left under magnetic stirring for 4 hours and nanoparticles formed, due to interactions taking place between TPP and ThioCHI, were isolated by lyophilization. Nanoparticles prepared were characterized by SEM for their morphology and by DLS for their size and surface potential.

Scanning electron microscopy (SEM) images were obtained with a JEOL 2011 electron microscope (JEOL Ltd., Tokyo, Japan). The prepared nanoparticles were coated with carbon, and images were collected by applying accelerating voltage 20 kV, probe current 45 nA, and counting time 60 s. EDX analysis was also performed.

### 2.5. Estimation of Cytotoxicity Effect NanoThioCHI-anti-VEGF

In order to assess the cytotoxic impact of nanoThioCHI-anti-VEGF to ASCs, after coculture of ASCs in augmented concentrations of NPs for 48 h, cell supernatant was removed, and MTT reactant (3-(4,5-dimethylthiazol-2-yl)-2,5-diphenyltetrazolium bromide) (Sigma) was introduced in a ratio of 1 : 10 in culture medium for a 4 h incubation in 37°C with 5% C*Ο*2. Upon the removal of the MTT, 200 *μ*l/well of DMSO was introduced for one additional hour of incubation in the same conditions. The reduction of MTT was calculated using Victor3TM Plate Reader (PerkinElmer, Massachusetts, USA) in 570-630 nm.

### 2.6. RVO Animal Model Induction and ASC/NP Transplantation

Twenty-four New Zealand rabbits were included in the study (*n* = 24). All rabbits weight approximately 4 kg and were 6 months old. The rabbits were kept in a room with a standard 12-hour light–dark cycle and had free access to food and water. All animals were acclimated to the facilities for 7 days prior to admission to the experimental sessions. Before the study, a complete and thorough ophthalmic examination was performed in all rabbits to ensure that they were free of any ocular pathologic conditions.

The rabbits were randomly divided into the four following groups: group I ASCs (*n* = 6), group II ASCs + nanoThioCHI-anti-VEGF (*n* = 6), group III RVO (*n* = 6), and group IV control (*n* = 6).

All animals were anaesthetized with dexmedetomidine (Dexdomitor, Zoetis Hellas) 0.080-0.1 mg/kg b.w., intramuscularly, and ketamine (Imalgene 1000, Merial, France) 15 mg/kg b.w., intramuscularly. Also, 1-2 drops of topical anaesthetic (proxymetacaine hydrochloride, Alcaine, Alcon Laboratories Hellas) were installed. The ocular surface and the conjunctival fornix underwent sterile preparation for intravitreal injection; they were cleansed and disinfected with a mild antiseptic solution containing aqueous 0.5% povidone–iodine. Following sterile cleansing of the eye, a 27-gauge needle was inserted into the midvitreous ~3 mm posterior to the limbus in the superior/temporal quadrant.

For RVO induction, MEK kinase inhibitor, PD0325901 (CAYMAN), was used in a concentration calculated according to our cytotoxic assay results on a cellular-induced model of RVO [8]. In groups I-III intravitreal (iv), injection of dissolved in BSS (1 mg/eye) PD0325901 in a final volume of 100 *μ*l per eye was performed, while in group IV, only 100 *μ*l BSS was injected.

All animals received tobramycin (Tobrex eye drop solution, Alcon Laboratories Hellas) every 6 hours for the first postoperative day, and meloxicam (Metacam, Boehringer Ingelheim, Germany) was given for 3 days (0.2 mg/kg b.w., subcutaneously, SID).

12 days later, animals received as follows: group I, 2 × 10^6^ ASCs in 0.1 *μ*l BSS; group II, 2 × 10^6^ ASCs +5 mg/ml anti-VEGF nanoThio-CHI NPs; and group III-IV, BSS. One donor-derived ASC prepared cell line was detached from plastic surfaces mechanically by applying mild scratching to above the usage of any enzyme probably toxic during administration. The animals were sacrificed 2 weeks following injection. For euthanasia, a mixture of dexmedetomidine (0.1 mg/kg b.w., intramuscularly) and ketamine (15 mg/kg b.w., intramuscularly) followed by a high dose of iv propofol and potassium chloride was used. Enucleation was performed, and the eye globes were fixed for histopathological analysis or washed in PBS before RNA isolation for molecular analysis.

Before all the above, a pilot experiment was performed aiming to test the ability of PD0325901 to cause retinal damage and also to determine the appropriate time for stem cell transplantation. Eight New Zealand rabbits were included in this pilot study (*n* = 8) which is also approved by the Committee of the Department of Veterinary Medicine of Thessaloniki (632937(3368), 28450(102)/16.01.2019). The animals divided in two equal groups of four and under the same mentioned conditions anaesthetized and received as follows: control group, 100 *μ*l BSS, and group RVO, 1 mg/eye PD0325901 in a final volume of 100 *μ*l. 12 days later, all the animals were sacrificed and their eye globes were removed and subsequently immersed in 10% formaldehyde for histological analysis. Vitreous fluid collected for secreted factors' quantification as described below.

### 2.7. Clinical Evaluation

A thorough ophthalmological examination was conducted in all rabbits two days after RVO induction and then weekly for the rest of the follow-up period. The eyes of the animals were evaluated with the use of a portable slit lamp biomicroscope (Kowa Optimed Inc., Torrance, CA) for opacification, intraretinal hemorrhages and areas of retinal detachments, and common expected findings, as the histological examinations were expected to reveal.

For IOP evaluation, one drop of 0.5% ALCA INE (proparacaine hydrochloride 0.5%) was instilled in the eyes, and an electronic applanation tonometer (Tonopen VET) was used. Ophthalmoscopy was conducted with the use of PanOptic ophthalmoscope for evaluation of hemorrhages, detachments, and retinal disorganization.

### 2.8. Secreted Factor Quantification

**Figure 1:**
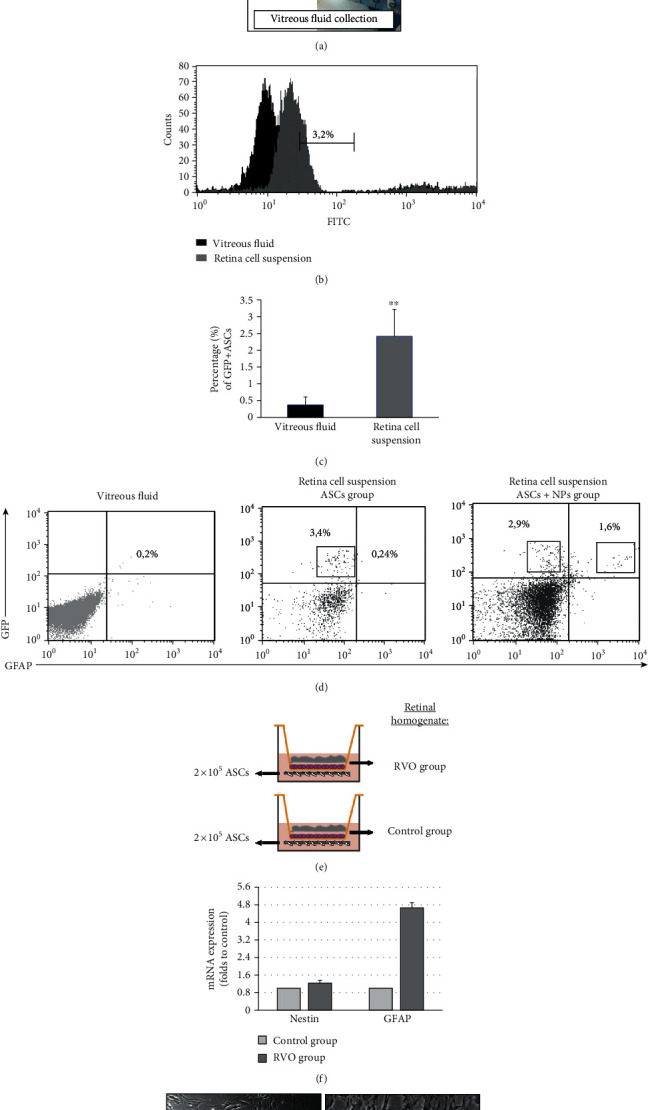
Detection of administered GFP+ ASCs in dissociated retina tissues and *in vitro* differentiation capacity of ASCs towards neural cells as a response to specific tissue stimuli in culture. (a) The muscles and connective tissues of the rabbit eyes were removed from the eye ball; the anterior segments of the eye including the cornea, iris, and lens were carefully removed. Then, the eye cup was used for retina isolation after sclera removal. Vitreous fluid is collected with a syringe. (b) Flow cytometry histogram presents the expression of GFP signal from retina cell suspension in comparison with vitreous fluid cell content. (c) Percentage of GFP+ cells in retina cell suspension and vitreous fluid. (d) Detection of GFAP+/GFP+ ASCs in retina cell suspension of the ASC + NP group. Data are expressed as the mean ± SD (^∗∗^*P* < 0.01 vs. RVO; *n* = 4). (e) Depiction of transwell culture systems with two types of retina homogenates. (f) Quantification of the transcriptional expression on neural markers NESTIN and GFAP in ASCs after culture in the presence of retina homogenates. (g) Morphological observation of ASCs after coculture with control (left) and RVO (right) tissue, respectively. Data are expressed as the mean ± SD (*n* = 2).

Peripheral blood was collected in anticoagulant CPD at days 0, 12, and 26 and centrifuged at 16000 g and 5 min for plasma isolation. Vitreous fluid was selected after puncture of the tissue with insulin syringe with 16G needle after tissue removal (Figure 1(a)). In the above biological samples, the secreted levels of both proinflammatory cytokines (interleukin 6 and TNF-alpha), vascular endothelial growth factor (VEGF), and soluble endothelial protein C receptor (sEPCR), a common RVO-related marker, were measured using sEPCR ELISA kits (CUSABIO).

### 2.9. Histological Analysis and Immunohistochemistry

At the end of the last follow-up examination, the rabbits were euthanized. Enucleation was performed, and the eye globes were immersed in 10% formaldehyde 37% solution for 16 to 24 h and then dehydrated in a series of graded alcohol solutions in the next 24 to 48 h prior to paraffin embedding. Blocks were obtained from cuts through the whole globe oriented perpendicular to the medullary wings. Sections 5 *μ*m thick obtained by a microtome were stained with Hematoxylin and Eosin (H&E) and Masson's trichrome staining and examined by light microscopy. Light-microscope images were photographed, and the thickness of inner nuclear layer (INL) and outer nuclear layer (ONL) was measured every 500 *μ*m from optic nerve head, only in regions without total disorganized retinal structure.

Immunohistochemistry of paraffin-embedded sections (3 *μ*m) was performed with glial fibrillary acidic protein (GFAP, 1 : 200; Sigma), ki-67 (MIB1, Clone TEC-3, 1 : 20; DAKO), and factor VIII (FVIII, 1 : 800; Abcam) antibodies. Bound antibodies were visualized by Dako Real Envision Detection System Peroxidase/DAB+, and slides were then studied by light microscopy. Mean of positive cells was calculated as the average of the measurements of five independent fields (400x).

### 2.10. Real-Time PCR Analysis

Total RNA was isolated from eye tissues collected in NucleoProtect RNA solution (MACHEREY-NAGEL) using the RNA isolation Nucleospin RNeasy Mini Kit (MACHEREY-NAGEL), according to the manufacturer's instructions. RNA concentration and purity analyzed using a NanoDropND-1000 UV-Vis Spectrophotometer. The expression related to the occlusion (VEGF, Apelin, Aqp4), inflammation (IL-6, Icam, IL-1b), and molecular pathways is responsible for RVO (PROCR, Pax6) and regeneration (GFAP) genes. 50 ng of total RNA was subjected to reverse transcription and qPCR using the KAPA SYBR® FAST one step qPCR Master Mix (2x) Kit. Reactions were performed using the PCR cycler (Rotor Gene 6000). Relative quantification was determined using the comparative C(t) method with normalization to the housekeeping gene GAPDH (glyceraldehydes 3-phosphate dehydrogenase). The oligonucleotide sequences of the primers designed (LabSupplies Scientific) are listed in Table 1. Each assay point was performed in triplicate.

### 2.11. Transwell System Culture Assays

To investigate the differentiation capacity of infused ASCs to retinal cells in vitro, 2 × 10^5^ ASCs were placed in the lower chambers of transwell culture systems and after their attachment separated by a permeable membrane (pore size 0.4 *μ*m; Corning Costar) from retinal homogenates (20 mg) derived from the RVO and control group, respectively, which are placed in the upper chambers. After 8-hour coincubation (37°C, 5% CO2), the cells were collected, RNA was extracted, and real-time PCR was performed in order to quantify the expression on neural markers NESTIN and GFAP as previously described with custom designed primers (Table 1).

### 2.12. Retina Isolation and Dissociation-Detection of Fluorescent Administered Differentiated ASCs with Flow Cytometry

Under microscope, the muscles and connective tissues of the rabbit eyes were removed from the eye ball; the anterior segments of the eye including the cornea, iris, and lens were carefully removed. Then, the eye cup was cut into 4 pieces from periphery to the optic nerve head. Each piece was carefully dissected into 3 parts: sclera, retinal pigment epithelium- (RPE-) Bruch's membrane-choriocapillaris complex (RBCC), and neurosensory retina which is used for further process (Figure 1(a)) [38].

Single retinas were minced and incubated in 1 ml of digestion solution (collagenase type II—Sigma—8 mg/gr retina) at 37°C for 30 min. Mechanical trituration was performed by pipetting 30 times every 10 min. Cells were centrifuged, at 600 g for 5 min at 4°C, and the digestion solution was removed. The cells were gently resuspended in 700 *μ*l of DMED including 10% FBS, passed through a 70 *μ*m cell strainer [39], and analyzed immediately with flow cytometry after staining with monoclonal antibody GFAP-phycoerythrin (PE) (EXBIO) for the detection of its coexpression with venus signal.

### 2.13. Statistical Analysis

The student *t*-test (unpaired, two-tailed) was used for the two-group comparisons including data as the mean ± standard deviation (SD). Differences were considered statistically significant when ∗*P* ≤ 0.05 or ∗∗*P* ≤ 0.01.

## 3. Results

### 3.1. Characterization of ASCs and Successful Genetic Modification

**Figure 2:**
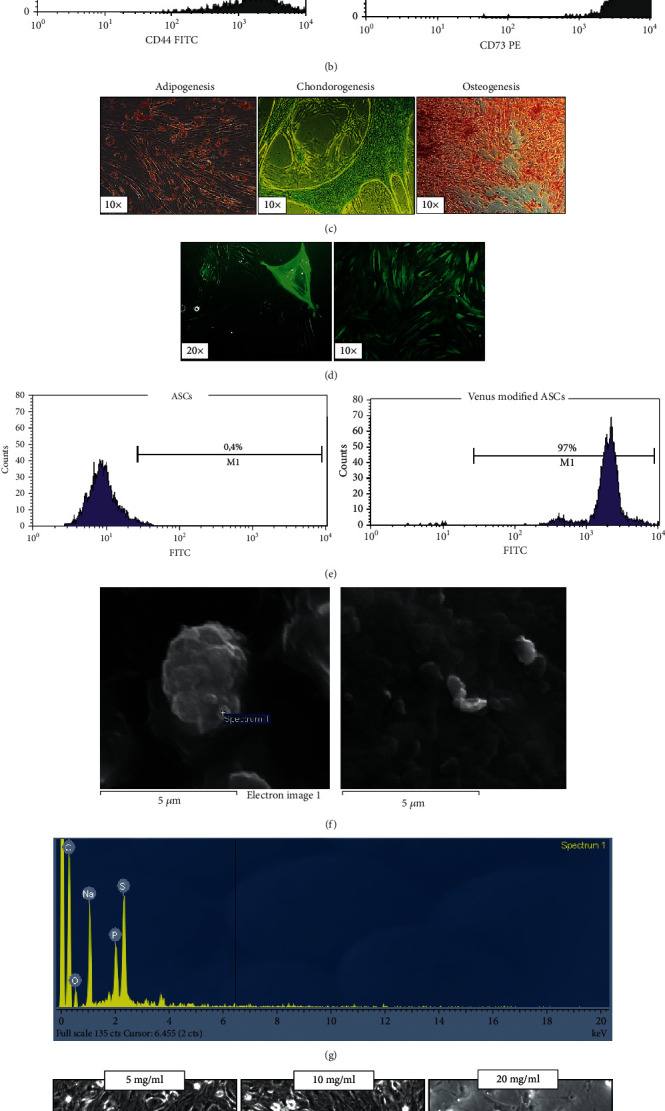
(a) Characterization of the expanded ASCs for administration with morphological observation of ASCs on passage 2 with approximately 95% confluency on plastic surface and (b) immunophenotypical characterization of ASCs for specific surface markers revealed their stem cell origin. (c) Morphological depiction of ASC differentiation capacity towards adipocytes, chondrocytes, and osteocytes after successful oil red, alcian blue, and alizarin red staining, respectively. (d) Genetic modified ASCs 24 h (left) and 1 month (right) after electroporation. (e) Flow cytometry analysis of venus positive ASCs before (left) and after (right) genetic modification. (f) NanoThioCHI-anti-VEGF characterization and determination of concentration for administration based on their cytotoxicity effect on ASCs in coculture with SEM photos of nanoThioCHI-anti-VEGF and (g) EDX analysis. (h) Images (20x) depict coculture of NPs with ASCs after 48 h (up); 5 mg/ml of NPs was selected as a safe concentration to avoid agglomeration and cytotoxic influence on coadministered ASCs (down). Data are expressed as the mean ± SD (^∗^*P* < 0.05 vs. control; *n* = 3).

ASCs after expansion in culture presented a typical fibroblast-like morphology (Figure 2(a)) while their typical mesenchymal immunophenotype was confirmed by flow cytometry for the high expression of surface markers CD105, CD90, CD44, and CD73 (Figure 2(b)). The ability of ASCs to differentiate into adipocytes, osteocytes, and chondrocytes was confirmed after positive stainings with the appropriate dyes (Figure 2(c)). Genetic modified ASCs expressed the green fluorescence dye especially 1 month after selection of GFP+ cells with G418 (Figure 2(d)) when the percentage of genetic modified cells is approximately 97% (Figure 2(e)).

### 3.2. NanoThioCHI-anti-VEGF Characterization and Determination of Their Administered Concentration

SEM photos are showed in Figure 2(f). As can be seen, nanoparticles prepared were smooth in surface, but agglomeration was observed ought to neutralization process during their formation. A closer look (Figure 2(f), right) showed that nanoparticles formed were spherical in shape with their sizes varying between 300 and 400 nm, while agglomeration is also obvious in this photo. EDX analysis showed the presence of sulfur atoms (S) mainly ought to ThioCHI than anti-VEGF due to its low concentration in nanoparticles to promote its time-controlled release upon injection (Figure 2(g)). A medium concentration of 5 mg/ml prepared nanoparticles was chosen for administration in order to avoid high agglomeration with simultaneous low cytotoxicity impact on coadministered ASCs (Figure 2(h)).

### 3.3. Evaluation of RVO Induction

12 days after PD0325901 intravitreal injections, representative images of H&E-stained retinas present retina disorganization and detachment of photoreceptors layer, intense hemorrhages, and formation of new vessels in the RVO group in comparison with normal retinal tissue where no signs of disorganization observed (Supplementary Figure 1A). At the same time, quantification of secreted levels of factors associated with pathologic neovascularization (VEGF) and RVO development (EPCR) in vitreous fluid showed significant increase confirming the successful development of the disease (Supplementary Figure 1B). Similar changes in transcriptional level also verify RVO model installation (data not shown).

### 3.4. Clinical Evaluation

**Figure 3:**
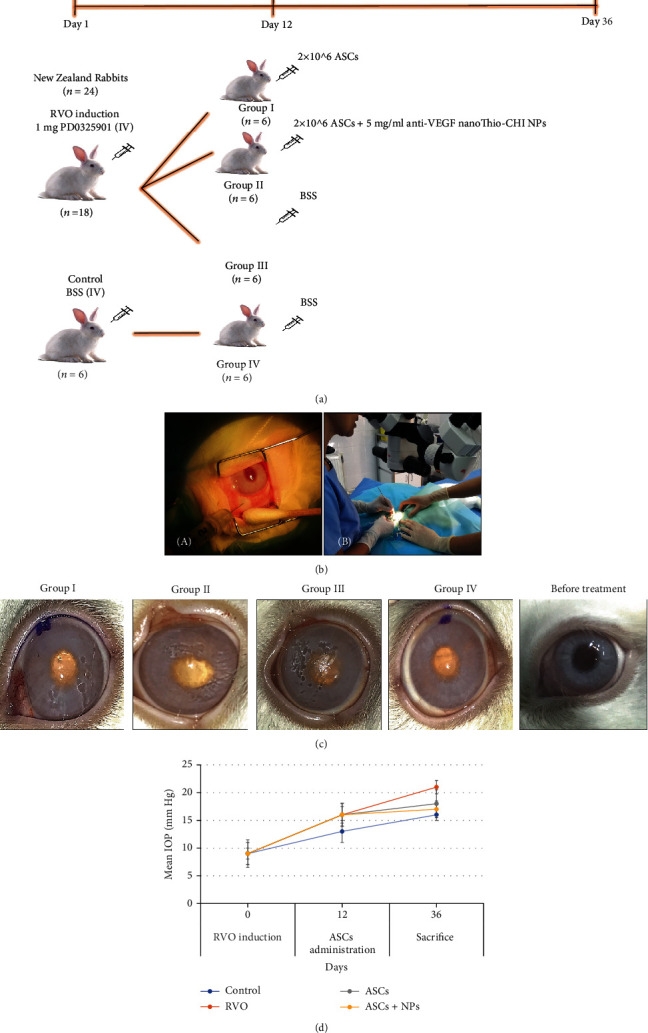
(a) Experimental design of the study. (b) Photo of the intravitreal injection of PD0325901 for RVO induction (day 1) (left) and of the surgery during stem cell administration (day 12) (right). (c) External photos during clinical examinations for the detection of any abnormalities in all groups and untreated eyes reveal retinal detachment in the RVO group which is limited in ASCs and especially in the ASC + NP group. (d) Evaluation of IOP in all groups shows an almost stable image after any treatment proving the safety of both RVO-animal model induction and ASC-related therapy. Data are expressed as the mean ± SD, and the IOP alterations are not statistically significant.

The experimental design of the study is presented in Figure 3(a). Up to the end of the experiment, no animal presented severe problems as manipulation result while all the procedures took place under totally sterile conditions (Figure 3(b)).

In day 2, groups I, II, and III presented multifocal or extensive retinal hemorrhages while moderate and diffuse retinal detachments were observed and, in some cases, liquification of vitreous. Similar image was observed 10 days later (day 12) before ASC administration.

10 days after ASC or ASC + NP injection (day 22), group III had still extensive retinal hemorrhages and detachments, retinal degeneration, and retinal vasculature attenuation along with liquification of vitreous. In one animal, cataract was also developed. Groups I and II showed no liquification of vitreous and mild-focal hemorrhages. During the last clinical evaluation (day 36), group III had pretty much the same appearance, while in group I a few focal hemorrhages and in group II no hemorrhages were noticed (Figure 3(c)). Ophthalmological evaluation of the rabbit eyes showed in all groups a slight IOP elevation the first days after injection but within the normal limits as a response to the intravitreal injections. However, neither PD0325901 nor ASCs (combined or not with NPs) caused statistically significant increase (Figure 3(d)).

### 3.5. Secreted Factor Quantification in Vitreous Fluid Reveals Successful RVO Induction and Beneficial Influence of ASC Combination with NanoThioCHI-Anti-VEGF

**Figure 4:**
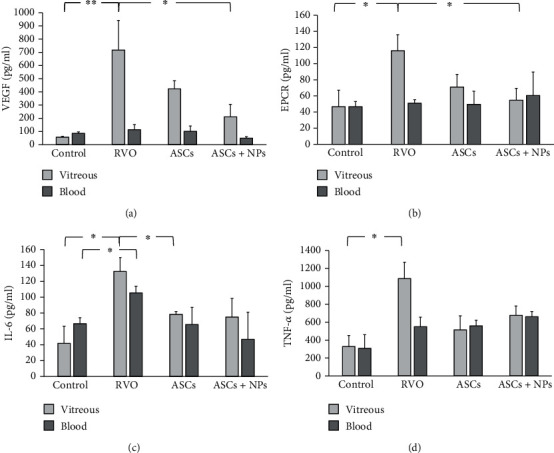
Quantification of secreted levels of factors associated with (a) pathologic neovascularization, (b) RVO, and (c, d) inflammation in vitreous fluid and peripheral blood of animals from all groups. Data are expressed as the mean ± SD (^∗∗^*P* < 0.01, ^∗^*P* < 0.05; *n* = 2).

The successful produce of RVO model was confirmed with the detection of statistically significant high levels of secreted VEGF in the RVO group (∗∗*P* = 0.007) (Figure 4(a)) accompanied with also increased levels of soluble EPCR (∗*P* = 0.03) (Figure 4(b)), in vitreous fluid. 2 weeks after proposed therapeutic regiment administration, the secreted levels of the above factors decreased, especially in ASCs + NPs (∗*P* = 0.03 for VEGF and ∗*P* = 0.02 for EPCR). VEGF and EPCR levels remained almost stable in animals' peripheral blood without significant changes. The levels of proinflammatory cytokines IL-6 and TNF-*α* in vitreous fluid presented analogous image with significant augmentation, as damage response (∗*P* = 0.03 for IL-6 and ∗*P* = 0.02 for TNF-*α*) and subsequent decrease after ASC or ASC + NP injection (Figures 4(c) and 4(d)). IL-6 secretion levels appeared the same fluctuation also in peripheral blood with great increase upon RVO induction (∗*P* = 0.04) following of reduction in therapy groups.

### 3.6. The Translational Profile of Retina Tissues Confirms Intense Response to Injections

**Figure 5:**
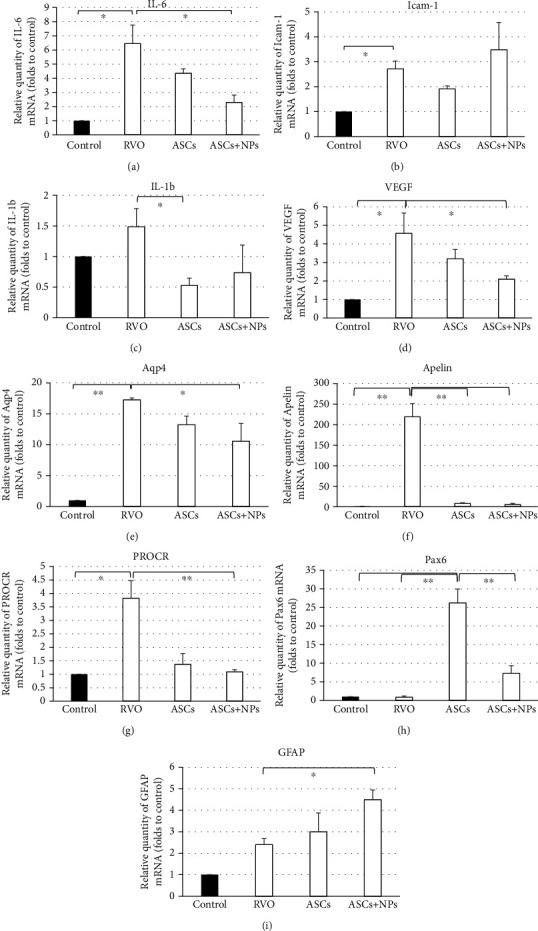
Expression of RVO, inflammation, and regeneration-related genes in all groups as was evaluated by real-time PCR analysis 2 weeks following transplantation. Data are expressed as the mean ± SD (^∗∗^*P* < 0.01, ^∗^*P* < 0.05 vs. control; *n* = 3).

The analysis of the translational profile via qPCR confirmed the inflammation as a result of RVO induction which is presented with observed high expression levels of IL-6 (∗*P* = 0.02), Icam-1 (∗*P* = 0.04), and IL-1b genes (Figures 5(a) and 5(c)). At the same time, genes involved in RVO development such as VEGF, Aqp4, Apelin, and PROCR showed elevated expression in the RVO group (∗*P* = 0.03, ∗∗*P* = 0.003, ∗∗*P* = 0.001, and ∗*P* = 0.03, respectively) (Figures 5(d)–5(g)).

Aiming the determination of ASC or ASC + NP transplantation effect on PD032901-treated animals, mRNA levels of all the previously mentioned genes were quantified too (Figures 5(d)–5(g)). Although proinflammatory cytokine expression is limited in some cases statistically significant (IL-6, ∗*P* = 0.04 in the ASC + NP group; IL-1b, ∗*P* = 0.03 in the ASC group), all related to RVO genes are remarkably decreased especially in the ASC + NP group (VEGF, ∗*P* = 0.03; Aqp4, ∗*P* = 0.045; Apelin, ∗∗*P* = 0.002; PROCR, ∗∗*P* = 0.008). The initiation of retina repair, involving cellular regeneration for restoration, was confirmed with surprisingly high translational levels of Pax6 and GFAP in the ASC and ASC + NP group, respectively, in comparison with RVO corresponding levels (∗∗*P* = 0.005, ∗*P* = 0.04).

### 3.7. Histological Assessment

**Figure 6:**
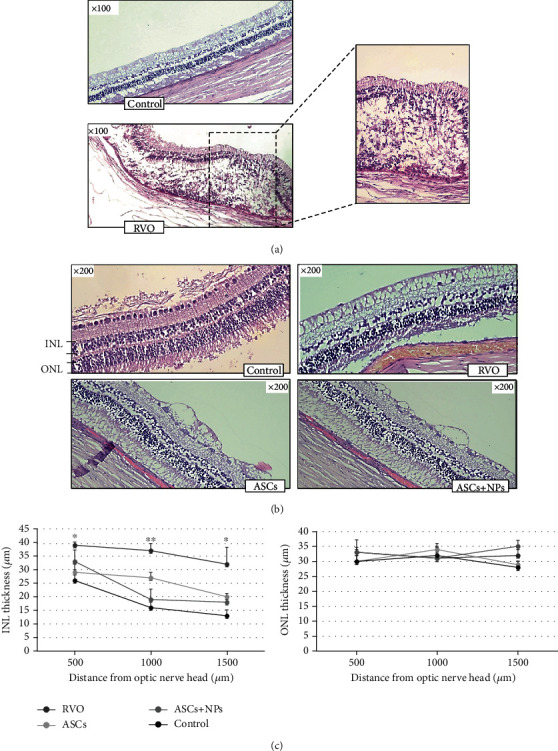
Representative images of H&E-stained retinas presenting: (a) retina disorganization and detachment in the RVO group; (b) the augmentation of INL in the RVO group and its following decrease in the ASC and ASC + NP groups. (c) Plots below illustrate quantitative INL and ONL thickness data. Data are expressed as the mean ± SD (^∗∗^*P* < 0.01, ^∗^*P* < 0.05 vs. control; *n* = 2).

Representative images of H&E-stained retinas showed retina disorganization and detachment in the RVO group in comparison with absolute normal architecture which observed in the control group despite the BSS injection. The degeneration was characterized by multifocal-to-diffuse disorganization of the retinal layers with loss of nuclei from the outer and inner nuclear layers and collapse of the photoreceptor layer (Figure 6(a)). Although hemorrhagic perfusion, intracellular edema, and partial ganglion cell layer degeneration are still remaining in the ASC and ASC + NP groups, INL thickness, measured in sites without significant detachment, was surprisingly decreased in contrast to the RVO group reaching almost normal levels (Figures 6(b) and 6(c)).

**Figure 7:**
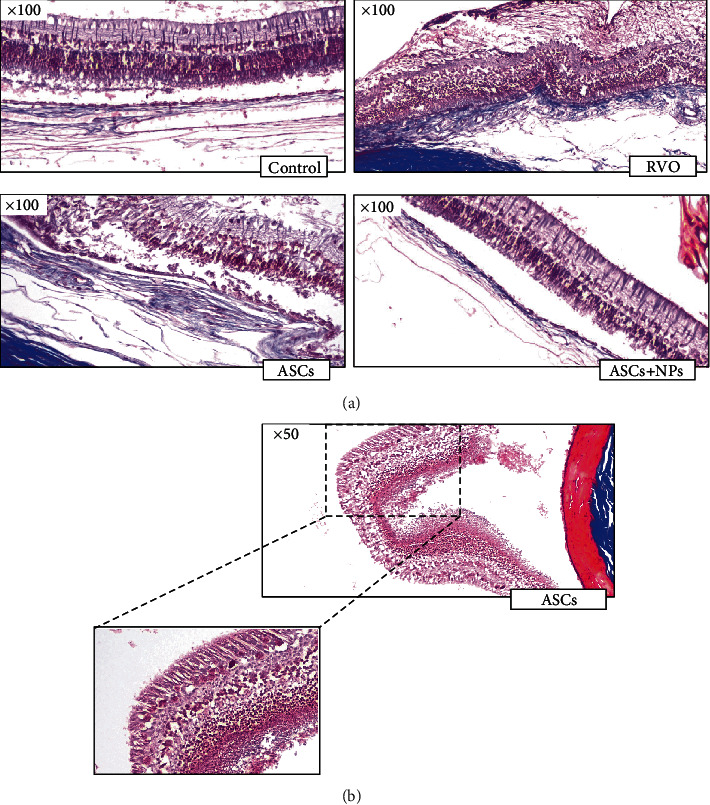
Masson trichrome stain. (a) Retina total degeneration and disorganized chorioretinal adhesion in the RVO group vs. the ASC groups where limited detachment is observed. (b) Müller and microglial cell migration to ASC intravitreal injection site leads to retina fold formation.

Masson trichrome stain also revealed retina total degeneration and disorganized chorioretinal adhesion in the RVO group vs. ASC groups where limited detachment is observed (Figure 7(a)). The formation of a folding retina fold only in ASC-administered groups probably present Müller and microglial cell migration capacity to ASCs' intravitreal injection site (Figure 7(b)).

### 3.8. Immunohistochemistry Observations

**Figure 8:**
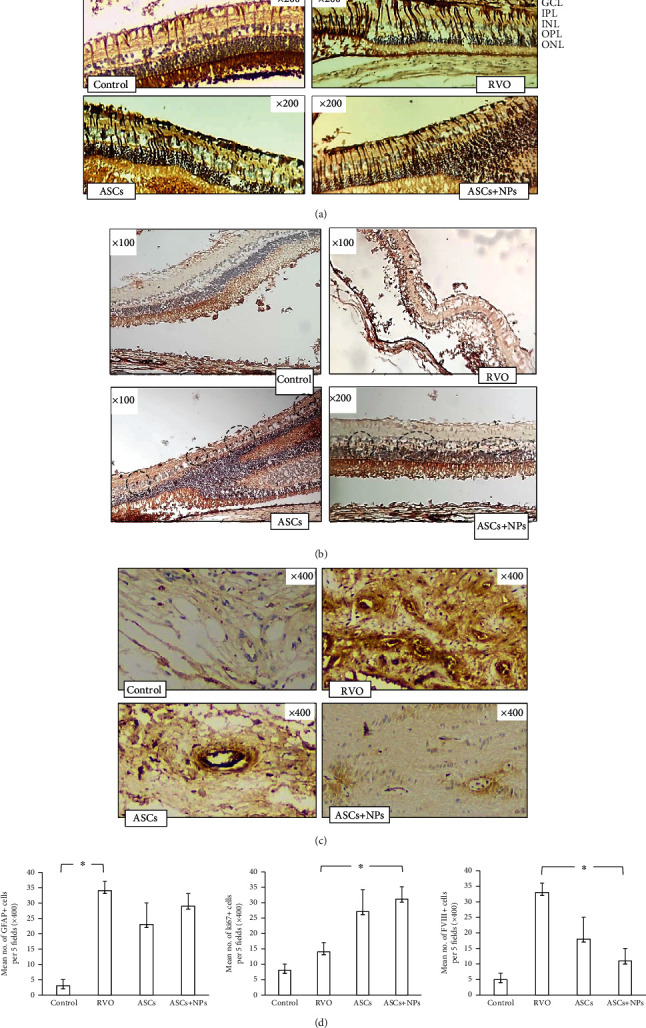
Immunohistochemistry staining of retina sections for GFAP, k-i67, and factor VIII reveals. (a) Intense response to damage with high GFAP secretion is confirmed with positive GFAP staining observed in the RVO group as a result of cell activation followed by subsequent decrease of positive signal at the height of the ganglion layer in the treatment groups. (b) Increased cell proliferation for tissue cellular restoration is estimated by counting several ki-67+ cells in the treatment groups (in five randomly selected fields) in relation to the control group and RVO group. (c) Limitation of pathological neovascularization (FVIII staining) is presented with decreased platelet concentration on injured vessels and counting of multiple pathological new vessels in the RVO group (in five randomly selected fields) in relation to the control group with important limiting of their number in the treatment groups. (d) Mean of positive cells per five fields. Data are expressed as the mean ± SD (^∗^*P* < 0.05; *n* = 2). GCL: ganglion cell layer; INL: inner nuclear layer; IPL: inner plexiform layer; ONL: outer nuclear layer; OPL: outer plexiform layer.

GFAP is localized in control retinas to astrocytes and to some Müller cell fibers in the ganglion cell and inner plexiform layers presenting almost negative staining for GFAP. In retinal sections obtained after RVO induction, Müller cell fibers were stained positive as they penetrate whole retina while GFAP positive staining was also observed in the ASC groups, especially in the ASC + NP group, with significant limitation on GCL demonstrating a hierarchy in the mode of cell regeneration as a response to stem cell transplantation (Figure 8(a)). The augmented mRNA GFAP levels, as described from qPCR, in combination with immunohistochemistry results in case of ASCs + NPs could possibly imply the presence of retina progenitor cells for tissue regeneration. This cell proliferation is also confirmed from ki-67 positive staining in INL of both the ASC and the ASC + NP groups combined with limitation of pathological neovascularization presented with decreased platelet concentration on injured vessels (Figures 8(b) and 8(c)). The quantification of all the above is presented as the mean of positive cells for each antigen and confirms the indications of tissue depictions (Figure 8(d)).

### 3.9. Administered GFP+ ASCs Detected in Contact with Retina Tissue Able to Transdifferentiated

Genetic modified GFP+ ASCs successfully detected in dissociated retina tissues (Figures 1(b) and 1(c)) revealing their closed contact with retina upon administration, something that is also confirmed from the characterized retina fold formation as we described previously (Figure 7(b)). All retina cell suspensions expressed an average of 2.4% GFP+ signal in a statistically significant way (∗∗*P* = 0.003) while ASCs are not detected in vitreous fluid. At the same time, the staining with GFAP, and remarkably only in case of the ASC + NP group, demonstrating a subpopulation of ASCs (GFP+) simultaneously able to differentiate in neural progenitors (GFAP+) maybe as a result of closed contact of stem cells with injured tissue enables the secreted factors stimulate the differentiation (Figure 1(d)). The in vitro simulation of ASCs contact with damaged tissue also led to mainly increased GFAP and fewer Nestin translational levels and morphological change (Figures 1(e) and 1(g)). The absence of GFAP expression in the ASC group in vivo demonstrates that time-controlled release of anti-VEGF could be able to promote ASC differentiation with a possible mechanism which enable their action due to neovascularization limitation.

## 4. Discussion

RVO is the fifth leading cause of blindness with the precise mechanisms of underlying pathophysiology and etiology requiring further elucidation [40–42]. Recent studies show that patients with RVO presented lower quality of life in comparison with controls [4]; thus, the proper use of the new diagnostic devices could contribute to a better visual outcome and QoL of affected individuals. Optical coherence angiography (OCTA) is a noninvasive diagnostic technique that has been introduced and widely used in recent years due to its advantages over the previous gold standard fluorescein angiography (FA). The OCTA provides the clinician with depth-resolved images of retinal and choroidal vessels, with better delineation of the foveal avascular zone and with dye independency. On the other hand, there is lack of normalized patient data, artefactual projection issues have emerged, and low-flow lesions or pathologic conditions cannot be easily detected with this technique. New OCTA platforms with appropriate algorithms have been developed in order to detect microvasculature of the retina that run in both spectral-domain (SD) and swept-source (SS) OCT machine [7]. Swept-source optical coherence tomography angiography (SS-OCTA) is also more precise in detecting the location and extent of maximum ischemic insult following RVO compared to FFA [8].

Anti-VEGF drugs intravitreally injected as innovative therapeutic approaches [43] while recent studies support the use of intravitreal dexamethasone implants in patients with retinal vascular diseases, especially in those with macular edema [3, 10–14]. Novel cellular therapies such as MSCs' application may be also useful due to the favorable effect of the paracrine action of these cells [44, 45].

ASCs can be easily isolated following a noninvasive procedure for tissue collection, and their use seems to be promising. However, several studies have reported significant complications after the injection of ASCs, leaving this kind of therapeutic approach on the sidelines. Kuriyan et al. reported a case series of three patients who underwent intravitreal injection of autologous ASCs for age-related macular degeneration (AMD) not regulated by the FDA [33]. A similar case of intravitreal stem cell injections at a different, unaffiliated stem cell clinic with blinding complications, including severe bilateral retinal detachments, has been recently reported [34] while another report documents a retinal detachment following intravitreal adipose tissue-derived stem cells with vision loss from 20/50 to hand motion vision [46–48].

It could be assumed that intravitreally injected ASCs may include autologous fibroblasts or cells that differentiate into autologous fibroblasts [49]. Intravitreal injection of autologous fibroblasts is a classic animal model of vitreoretinopathy and retinal detachment [50] and may explain the multitude of retinal detachments after intravitreal injection. In addition to the vision loss related to intraocular pressure and retinal detachment, vision loss may have developed because of a combination of factors, including toxic effects on the retina or optic nerve caused by the injected material, which may have included the enzymes used in the preparation [48].

We here administered an almost purified and totally immunophenotypically characterized population of ASCs, in similar to previously mentioned number of stem cells per eye, prepared for transplantation without enzymes treatment in order to avoid negative outcomes related to all the previously mentioned concerning the cellular product. Only in case of ASCs + NPs we surprisingly present ASC capacity to express in vivo neural markers as a response to their close contact with damaged tissue. Combining this with the significant decrease which observed in the levels of VEGF and EPCR in vitreous fluid and with the limited translational levels of IL-6, VEGF, Aqp4, Apelin, and PROCR genes, we here show for the first time the beneficial influence of anti-VEGF continuous release in ASCs' action.

Many studies using RVO animal models have been reported. However, these previously reported animal models have important limitations which despite of the lack of important clinical symptom exhibition and laser-induced inflammation in irradiated areas also include immediate spontaneous [36]. Especially in case of ASC-mediated treatments, the above limitation is vital given the duration required for the regenerative potential of these cells.

Huang et al. have proposed the intravitreal administration of PD0325901 to Dutch-Belted rabbits as a reliable preclinical experimental model of the disease [35]. Main clinical characteristics such as retinal vascular occlusion and vascular hemorrhage and leakage were observed in the rabbit model following PD0325901 treatment. In our pilot study, we showed that 12 days after PD0325901 injection animals present a typical histological retina disorganization characterized of photoreceptor detachments and significant hemorrhages, followed by increased levels of related to RVO secreted factors in vitreous fluid. The successful induction of the disease was vital for further research aiming to determine ASC effect on the installed damage. The results of the present study indicate that also in transcriptional, translational, and histological level the PD0325901 induction could satisfactorily promote the development of the disease which keeps the majority of histological and molecular characteristics up to 36 days after pharmaceutical induction. The mechanism underlying this procedure remains to be clarified. In addition, taking into consideration the already described appearance of clinical findings, including vascular leakage, capillary loss, retinal detachment, edema, and retinal vasculature attenuation in just 8 days upon PD0325901 injection [35], the early ASC therapeutic approach could be evaluated. In this work, we tried to evaluate the influence of the early administration of ASCs or their combination with nanocarriers in an animal model suitable for this purpose.

It has been referred that nanoparticles can successfully deliver antiangiogenic drugs for sustained and controlled release followed by targeted drug delivery with significant enhancement of their bioavailability [51, 52]. Polymeric nanoparticles are currently a promising candidate for ocular drug delivery because they are completely degradable, nontoxic, and easy to be functionalized with various types of drugs [53] while chitosan has already been used as a carrier for antibody delivery [54, 55].

Based on all the above, we here try for the first time *in vivo*, our novel anti-VEGF nanocarrier of chitosan modified with thiol groups aiming the increase of its adhesiveness on the administration environment for locally targeted drug release. As we have recently described, nanoThioCHI-anti-VEGF presents no cytotoxic effect upon addition in cell culture even in high concentrations while the respective anti-VEGF nanocarrier is capable of time-controlled and sustained release of the drug for 8 continuous days [22]. This *in vitro* time period is likely to correspond to an *in vivo* time period greater than the already referred to the existed literature [51]. Based on this evidence, this study suggests the *in vivo* combination of this long-term release of anti-VEGF from nanocarriers, limiting the disadvantages that accompany the administration of anti-VEGF-approved drugs and occur due to the frequent required infusions, with ASCs aiming the preparation of a limited-neovascularization microenvironment to enable ASC beneficial effect.

Conclusively, we here propose the combination of increased adhesiveness anti-VEGF nanocarriers with a purified expanded and fully characterized ASCs' population as a potential therapeutic regimen against RVO and other related to retinal degeneration diseases by using an easily pharmaceutically induced-RVO animal model. The mechanism underlying this combinative positive result must be further studied.

## Figures and Tables

**Table 1:** Primers used in qPCR.

Primers	Sequence
VEGF F	TGGTCCCAGGCTGCAC
VEGF R	TCGATCGGCTGGCAGT
Apelin F	CACCTCGCACCTGCTGTA
Apelin R	GAACGGGAATCATCCAAAC
Aqp4 F	GAGTATGTCTTCTGTCCTG
Aqp4 R	ACGGTCAATGTCAATCAC
IL-6 F	CTACCGCTTTCCCCACTTCAG
IL-6 R	TCCTCAGCTCCTTGATGGTCTG
Icam F	CGCTGTGCTTTGAGAACTGTG
Icam R	ATACACGGTGATGGTAGCGGA
IL-1b F	TGCAGGAGCTTTGGGATTCT
IL-1b R	CAGCTCATACGTGCCAGACA
PROCR F	GATCTGTTTCTCCCCGCGAT
PROCR R	CGAGGGCTGATGAGAAGTCC
Pax6 F	AGCTCCAATGGCGAAGACTC
Pax6 R	GCGCTGTACGTGTTTGTGAG
GFAP F	GTACCAGGACCTGCTCAAT
GFAP R	CAACTATCCTGCTTCTGCTC
Nestin F	CAGCGTTGGAACAGAGGTTGG
Nestin R	TGGCACAGGTGTCTCAAGGGTAG
GAPDH F	TGACGACATCAAGAAGGTGGTG
GAPDH R	GAAGGTGGAGGAGTGGGTGTC

## Data Availability

The data used to support the findings of this study are available from the corresponding author upon request.

## Supplementary Materials

Supplementary Materials Supplementary Figure 1: (A) Representative images of H&E-stained retinas presenting: normal retinal tissue without signs of disorganization in control group (up) and retina disorganization and detachment of photoreceptor layer, intense hemorrhages, and formation of new vessels in the RVO group (down). (B) Quantification of secreted levels of factors associated with pathologic neovascularization (VEGF) and RVO development (EPCR) in vitreous fluid 12 days after PD0325901 administration. Data are expressed as the mean ± SD (∗∗*P* < 0.01; *n* = 2).
