# Supplementary figures and images for: Intravitreal Administration Effect of Adipose-Derived Mesenchymal Stromal Cells Combined with Anti-VEGF Nanocarriers, in a Pharmaceutically Induced Animal Model of Retinal Vein Occlusion
Source: Stem Cells Int. 2022 Feb 23;2022:2760147. doi: 10.1155/2022/2760147 (PMC8890865; doi:10.1155/2022/2760147)

**A**

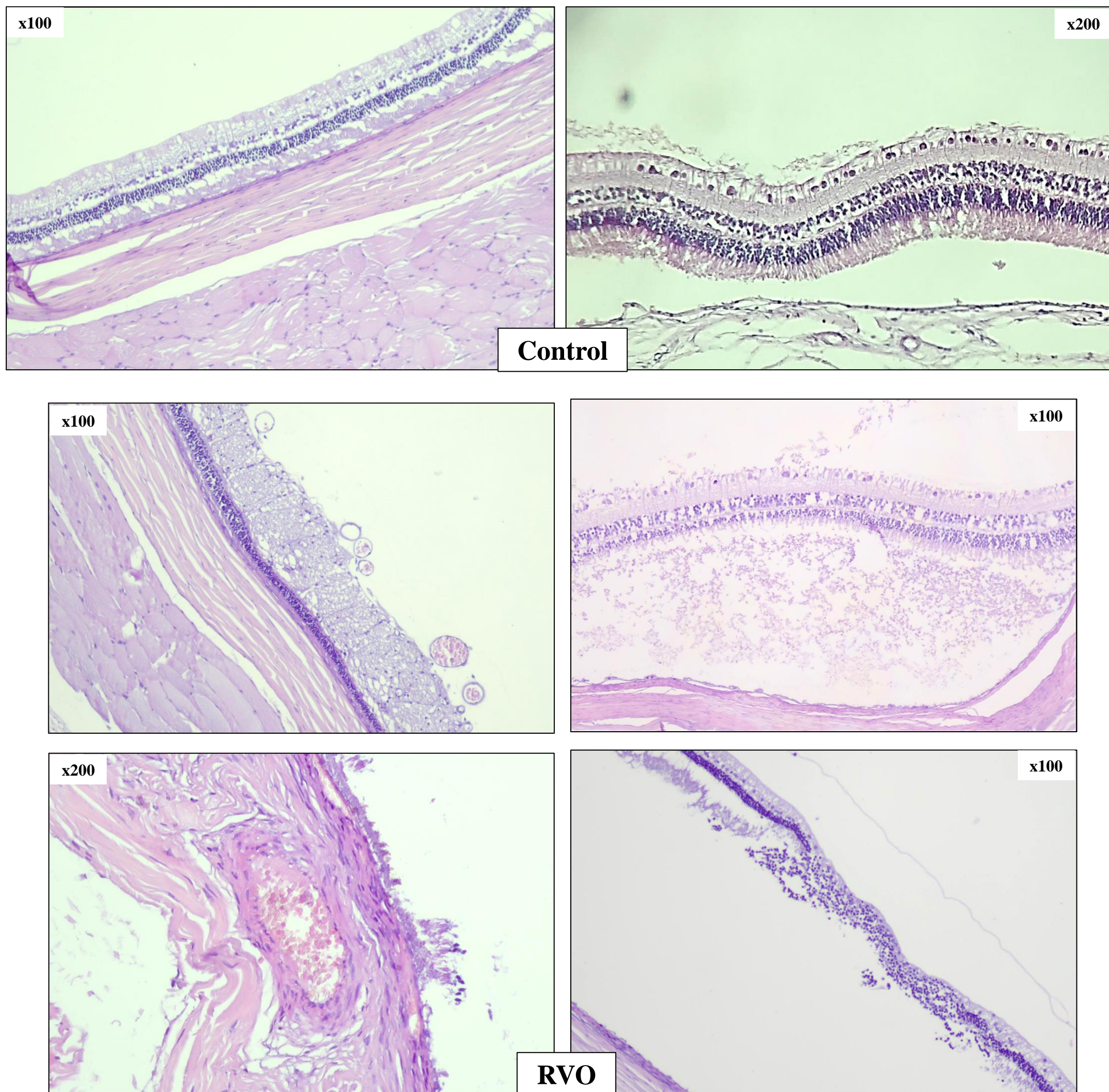

**B**

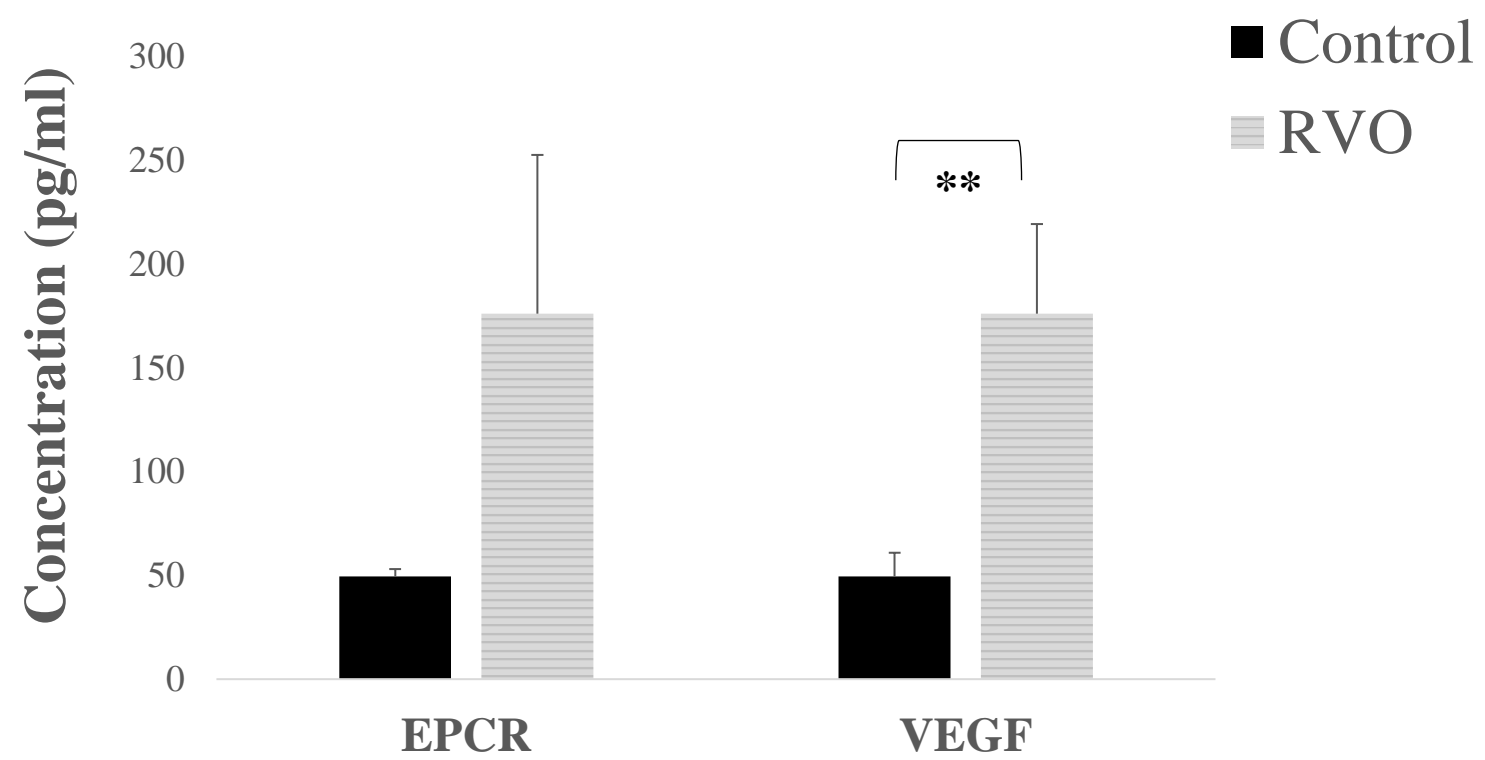

Supplement: Supplementary Materials — Supplementary Figure 1: (A) Representative images of H&E-stained retinas presenting: normal retinal tissue without signs of disorganization in control group (up) and retina disorganization and detachment of photoreceptor layer, intense hemorrhages, and formation of new vessels in the RVO group (down). (B) Quantification of secreted levels of factors associated with pathologic neovascularization (VEGF) and RVO development (EPCR) in vitreous fluid 12 days after PD0325901 administration. Data are expressed as the mean ± SD (∗∗P < 0.01; n = 2). [file 2760147.f1.pdf]
